# Enhanced Cytotoxicity on Cancer Cells by Combinational Treatment of PARP Inhibitor and 5-Azadeoxycytidine Accompanying Distinct Transcriptional Profiles

**DOI:** 10.3390/cancers14174171

**Published:** 2022-08-28

**Authors:** Tomonori Araki, Kensuke Hamada, Aung Bhone Myat, Hideki Ogino, Kohei Hayashi, Miho Maeda, Ying Tong, Yasufumi Murakami, Kazuhiko Nakao, Mitsuko Masutani

**Affiliations:** 1 Department of Molecular and Genomic Biomedicine, Center for Bioinformatics and Molecular Medicine, Nagasaki University Graduate School of Biomedical Sciences, Nagasaki 852-8523, Japan; bb55318002@ms.nagasaki-u.ac.jp (T.A.); aumiat.abm@nagasaki-u.ac.jp (A.B.M.); bb55321035@ms.nagasaki-u.ac.jp (K.H.); bb55320022@ms.nagasaki-u.ac.jp (Y.T.); 2 Department of Gastroenterology and Hepatology, Nagasaki University Graduate School of Biomedical Sciences, Nagasaki 852-8501, Japan; kazuhiko@nagasaki-u.ac.jp; 3 Biochemistry Division, National Cancer Center Research Institute, Tokyo 104-0045, Japan; ken-hamada@taiho.co.jp (K.H.); gynohyde@gmail.com (H.O.); mihoh91329@gmail.com (M.M.); 4 Department of Biological Science and Technology, Faculty of Industrial Science and Technology, Tokyo University of Science, Tokyo 162-8601, Japan; yasufumi@rs.noda.tus.ac.jp; 5 Medical Research Institute, Tokyo Medical and Dental University, Chiyoda-ku, Tokyo 113-8510, Japan

**Keywords:** PARP inhibitor, 5-aza-2′-deoxycytidine, microarray, transcription, DNA methylation

## Abstract

**Simple Summary:**

We investigated the effect of combinational use of PARP inhibitors on cytotoxicity of 5-aza-dC in human cancer cell lines. The combinational treatment of 5-aza-dC and PARP inhibitor PJ-34 exhibited a stronger cytotoxicity compared with their treatment alone in blood cancer HL-60, U937, and colon cancer HCT116 and RKO cells. In microarray analysis, combinational treatment with PJ-34 and 5-aza-dC caused different broad changes in gene expression profiles compared with their single treatments in both HCT116 and RKO cells. The profiles of reactivation of silenced genes were also different in combination of PJ-34 and 5-aza-dC and their single treatments. The results suggest that a combination of 5-aza-dC and PARP inhibitor may be useful by inducing distinct transcriptional profile changes.

**Abstract:**

Poly(ADP-ribose) polymerase (PARP) is involved in DNA repair and chromatin regulation. 5-Aza-2′-deoxycytidine (5-aza-dC) inhibits DNA methyltransferases, induces hypomethylation, blocks DNA replication, and causes DNA single strand breaks (SSBs). As the PARP inhibitor is expected to affect both DNA repair and transcriptional regulations, we investigated the effect of combinational use of PARP inhibitors on cytotoxicity of 5-aza-dC in human cancer cell lines. The combinational treatment of 5-aza-dC and PARP inhibitor PJ-34 exhibited a stronger cytotoxicity compared with their treatment alone in blood cancer HL-60, U937, and colon cancer HCT116 and RKO cells. Treatment with 5-aza-dC but not PJ-34 caused SSBs in HCT116 cell lines. Global genome DNA demethylation was observed after treatment with 5-aza-dC but not with PJ-34. Notably, in microarray analysis, combinational treatment with PJ-34 and 5-aza-dC caused dissimilar broad changes in gene expression profiles compared with their single treatments in both HCT116 and RKO cells. The profiles of reactivation of silenced genes were also different in combination of PJ-34 and 5-aza-dC and their single treatments. The results suggest that the combinational use of 5-aza-dC and PARP inhibitor may be useful by causing distinct transcriptional profile changes.

## 1. Introduction

DNA damage, genomic, and epigenomic instability induce the activation of oncogenes and the inactivation of tumor suppressor genes [1]. This results in malignant transformation of cells and survival against cell death-inducing stimulation, and through these processes, tumor cells that have acquired malignancy are selected. Various epigenome dysregulations, including DNA methylation, are also induced in cancer cells [2].

The antitumor effect of 5-aza-2′-deoxycytidine (5-aza-dC), a DNA demethylating agent that targets DNA methyltransferases (DNMTs), has been investigated in myelodysplastic syndromes (MDS) [3,4,5]. However, there are many silenced genes whose expressions could not be restored by single treatment of cancer cell lines with 5-aza-dC. It has been reported that a histone deacetylase (HDAC) inhibitor, which increases histone acetylation modifications in combination with DNA demethylating agents, acts in a coordinated manner to induce the expression of silenced cancer suppressor genes [6,7,8,9]. However, silenced cancer-suppressor gene expressions are not often restored even by the combination of DNMT inhibitor and HDAC inhibitor. New methods are needed to enhance the normalization of epigenome and chromatin abnormalities in cancer cells.

The enzyme poly(ADP-ribose) polymerase (PARP), which catalyzes poly(ADP-ribosylation), is one of the dynamic post-translational modifications of proteins. PARP uses NAD+ as a substrate to polymerize ADP-ribose residues using acceptor residues of Glu, Asp, Thr, Ser, and Lys. Among the 17 PARP family members that have been identified, PARP-1, which is the most well characterized, is activated by DNA strand breaks and is involved in DNA repair processes [10], chromosome stabilization, as well as the regulation of centrosome function [11]. PARP inhibitors have been thus clinically used for causing synthetic lethality to cancer cells that show defect in homologous recombination repair of DNA, including *BRCA1/2*-deficient cells [12]. The combinational treatment with chemotherapeutic drugs such as cisplatin [13] have been shown to enhance the cytotoxic effects of PARP inhibitors.

On the other hand, PARP1 is also activated by just DNA, histones [14,15], and by the interaction with phosphorylated ERK2, which induces the transcription of the ELK-1 target gene *c-FOS* [16]. Other reports also showed the function of PARP1 in gene transcription [17,18] and epigenomic regulation [19,20]. There are known examples of PARP-1 function as gene coactivators and corepressors. PARP-1 is required for retinoic acid (RA)-induced transcription of retinoic acid receptor (RAR) β2 genes [21]. In the absence of RA, the upstream sequence of the RAR β2 is associated with a corepressor complex, an inactive mediator complex, and PARP-1. Upon the binding of RA to RAR, the mediator complex becomes active, dissociating the corepressor complex and replacing it with a coactivator complex that is composed of histone acetylases, through the action of PARP-1.

PARP-1 is also required as a coactivator for the NF-κB-dependent induction of iNOS and other transcription factors [22]. Although binding of NF-κB together with the histone acetylase p300/CBP (cAMP-response element-binding protein [CREB]-binding protein) to the NF-κB recognition sequence is not sufficient to activate the transcription from chromatin, when several lysine residues near the BRCT motif of PARP-1 are acetylated by p300/CBP, this stabilizes the interaction with the mediator complex and activates transcription [22]. It has also been reported that PARP-1 acts as a coactivator of TCF-4 (T-cell factor-4) and binds to TCF-4 and β-catenin [17]. Mutations in the adenomatous polyposis coli (*APC*) tumor suppressor gene cause the intracellular accumulation of β-catenin, which activates the transcription factor TCF-4 and leads to adenoma formation, a precursor lesion of colon cancer. It has been shown that PARP-1 may contribute to early carcinogenesis by binding to TCF-4 and enhancing its transcriptional activity. In Drosophila, it was revealed that PARP-1 regulates transcription through chromosome decondensation [23]. In PARP-1-deficient mutants, puff formation that was induced on salivary chromosomes is suppressed and gene expression on the puffs is greatly reduced [23]. This suggests that poly(ADP-ribosylation) is required for the induction of gene expression that is associated with puff formation on the giant chromosomes of Drosophila salivary gland cells.

It is also shown that during embryonic development, PARP inhibition causes broad changes in histone modification and disturbs the embryogenesis processes [24,25]. While mechanisms of anti-tumor effects have been reported for PARP inhibitors that target the inhibition of DNA repair as a point of action, anti-tumor effects targeting transcription, chromatin, and epigenomic regulations have not been fully elucidated, while the combination of PARP inhibitor with MEK inhibitors in cancer patients with *KRAS* mutations was reported to involve transcriptional changes [26].

During gene silencing during cancer development, DNA methylation appears to occur in concert with histone modifications. Since methylCpG-binding protein2 (MeCP2), which specifically binds to methylated CpG, recruits HDAC and histone methyltransferases, DNA methylation may occur first, followed by histone deacetylation [27,28]. The activation of gene expression by HDAC inhibitors often does not work for genes with methylated DNA near the promoter [6]. This suggests that transcriptional repression occurs in the order of DNA methylation followed by histone deacetylation [29], but the details are unclear. In any case, the combination of HDAC inhibitors and DNMT inhibitors has been shown in concert to induce new cancer-regulated genes and is expected to have therapeutic applications [7]. PAR is reported to cause the interference of DNA methyltransferase 1 (Dnmt1) activity and PARP inhibition induced DNA hypermethylation in several cell lines [30]. This PAR-caused DNA hypermethylation is expected to affect broad transcriptional profiles.

It is considered necessary to clarify the possibility of PARP inhibitors to enhance therapeutic effects of 5-aza-dC on tumor cells by modulating gene expression profiles. Therefore, in this study, we examined whether PARP inhibitors enhance cytotoxicity against human tumor cells of blood cancer and colon cancers in combination with the DNA demethylating agent 5-aza-dC. We further analyzed the mechanism of action, focusing on the effects on gene expression profiles. The effect was also compared with the effect of HDAC inhibitor trichostatin (TSA).

## 2. Materials and Methods

### 2.1. Cell Culture

The human HL60 cells were obtained from the National Cancer Center. The human histiocytic lymphoma U937 was obtained from American Type Culture Collection (Manassas, VA, USA). The human colon cancer cell lines RKO (American Type Culture Collection) and HCT116 (kindly provided by Dr. Bert Vogelstein (The John Hopkins University) were used. The culture medium was McCoy’s 5A Medium (GIBCO, Tokyo, Japan) or Dulbecco’s Modified Eagle’s Medium (GIBCO) for HCT116, Dulbecco’s Modified Eagle’s Medium (SIGMA) for RKO, RPMI1640 medium (GIBCO) for the chronic myelomonocytic leukemia cell line HL60 and U937 cell lines. Fetal bovine serum (10%) (Hyclone) and penicillin-streptomycin (GIBCO) were added, respectively. For cell counting, RKO and HCT116 were treated with trypsin-EDTA (GIBCO), then an equal volume of 0.4 % trypan blue was added and the viable cell count was determined on a blood cell counting plate.

### 2.2. Chemicals

We used DNA methylation inhibitor 5-aza-2′-deoxycytidine (5-aza-dC; Sigma-Aldrich, St. Louis, MO, USA), PARP inhibitors *N* (6-oxo-5,6-dihydro-phenanthridin-2-yl)-*N*,*N*-dimethyl-acetamide-HCl (PJ-34; ALEXIS Biochemicals), and olaparib and talazoparib (Selleck Biotech, Houston, MA, USA), and histone deacetylation inhibitor trichostatin A (TSA) (Sigma-Aldrich).

### 2.3. Flow Cytometry

The cells were harvested, washed with PBS (-), and transferred into ethanol/ PBS (-) (7:3, *v*/*v*) and fixed. After centrifugation, RNaseA/ PBS (-) was added to the cells at a final concentration of 1 mg/mL RNaseA for 30 min at 37 °C, followed by gentle shaking at 200× *g* for 5 min, and the cells were washed twice with 1 mL of PBS (-). The supernatant was filtered through a 200 µm nylon mesh, and propidium iodide (PI; Sigma-Aldrich) solution was added to a final concentration of 1 µg/mL and used for analysis with the FACSCalibur system (Becton-Dickinson, Mountain View, CA, USA).

### 2.4. Cytotoxicity Assay

For HCT116 cells, cell growth was measured by counting the viable cells using trypan blue staining or using the Cell Counting Kit-8 (CCK assay, Dojindo Laboratories, Tokyo, Japan) containing water soluble tetrazolium dye (WST-8), according to the manufacturer’s instructions. IC50 and IC30 values were also calculated with the CCK-assay.

### 2.5. Pulsed-Field Gel Electrophoresis

Single strand breaks (SSBs) and alkali-labile DNA were analyzed by pulse-field gel electrophoresis of genome DNA in alkaline conditions and after transferred to membrane, Southern blot hybridization with genomic DNA was carried out [31,32]. Briefly, after trypsin treatment, cells (1.5 × 10^5^ cells/plug) were counted and embedded in a 1% agarose plug. After proteinase K treatment at 50 °C for 20 h, they were replaced with wash buffer consisting of 50 mM EDTA (pH 8.0) and stored at 4 °C. To detect single-strand breaks, alkaline treatment was performed by immersing the samples in alkaline buffer (0.3 M NaOH, 1 mM EDTA) at room temperature for 1 h, followed by immersion in wash buffer (0.5 × TBE) for 1 h, and then the treated samples were incubated in 0.5 × TBE and applied to a 1% agarose gel consisting of 0.5 × TBE, electrophoresed for 20 h using CHEF MAPPER (BIO-RAD) with the conditions: Forward V-Gradient: 5.4 V/cm, Initial switch time: 5 s, Final switch time: 60 s, Reverse V-Gradient: 3.6 V/cm, Initial switch time: 5 s, Final switch Initial switch time: 5 s, Final switch time: 60 s. The samples were then stained with 0.5 µg/mL ethidium bromide and photographed. Southern blotting method was performed using ^32^P-dCTP-labeled human genomic DNA as a probe and analyzed using BAS-2500 (Fuji-Film Tokyo, Japan).

### 2.6. DNA Ladder Formation Induced by Apoptosis

After cell counting, the cells were suspended in lysis buffer (10 mM Tris-HCl (pH 7.4), 10 mM EDTA, 0.5% Triton-X100) and the fragmented DNA fraction was collected as supernatant, treated with RNase A and proteinase K at 37 °C, and was precipitated with 2-propanol. The DNA was dissolved in TE (pH 8.0) and electrophoresed with 2% agarose.

### 2.7. Genomic DNA Extraction and Restriction Enzyme Treatment

The cells were collected by trypsin treatment, gently suspended in cell lysis buffer (final concentration 20 mM Tris-Cl (pH 8.5), 25 mM EDTA, 0.5% SDS, 10 mM NaCl, 1 mg/mL proteinase K) in a wide-mouth tip and kept warm overnight at 55 °C. After phenol/chloroform extraction and ethanol precipitation, genomic DNA was dissolved in 100–200 µL of TE (pH 8.0). The DNA was digested with the methylation-insensitive restriction enzyme *Msp* I and the methylation-sensitive restriction enzyme *Hpa* II, an isoschizomer of *Msp* I, at 37 °C for 18 h. The DNA was also similarly digested with methylation- sensitive enzymes *Nae* I and *Sma* I. A quantity of 1 µg equivalent DNA was electrophoresed in a 1% agarose gel with Tris-acetate-EDTA buffer overnight, and the gels were stained with ethidium bromide.

### 2.8. Microarray Analysis

The cells were seeded 24 h after medium exchange including drug treatment, and collected 72 h after medium exchange. Based on the results of the condition study, seven conditions were set for drug treatment: 3 µM PJ-34, 0.5 µM 5-aza-dC, and 150 nM TSA for HCT116, and 2 µM PJ-34, 0.5 µM 5-aza-dC, and 150 nM TSA for RKO, with the three drugs alone, in combinations of two, and in combinations of three. A total of seven conditions were set. The control and solvent controls were also added to those conditions. RNA was extracted using Isogen (Nippon Gene, Tokyo, Japan), and 3 µg of total RNA that was recovered was used for cRNA synthesis of probes according to Affymetrix protocol. Then, the RNA was hybridized to Human Genome U133 Plus 2.0 (Affymetrix, Santa Clara, CA, USA). The data were imported into GeneSpring GX^®^ Software Database (Silicon Genetics, Redwood City, CA, USA) for analysis. The excel file of microarray data is attached as a supplemental Table S2.

### 2.9. Statistical Analysis

The data were expressed as the mean ± S.E. Statistical significance was indicated when the *p* value was less than 0.05. In this study, the data were analyzed using Tukey’s test or Mann–Whitney *U*-test. Synergistic effects were analyzed by two-way ANOVA.

## 3. Results

### 3.1. PARP Inhibitor Enhanced Cytotoxicity of 5-aza-dC in HL-60 and U937 Cells

**Figure 1:**
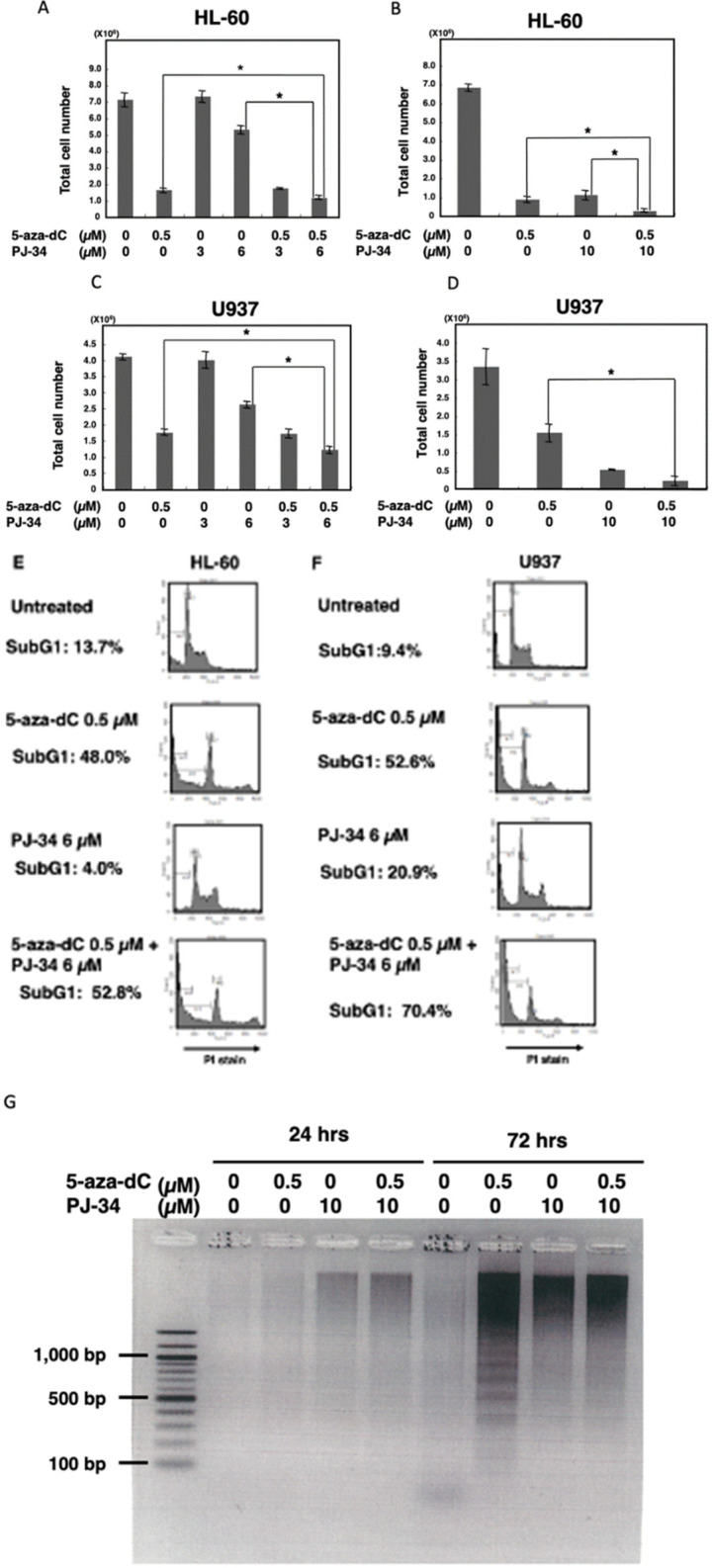
The effect of PJ-34 on the cytotoxicity of 5-aza-dC in HL-60, U937 cells. (**A**,**B**) HL-60 cells were treated with 5-aza-dC and PJ-34 or a combination of these chemicals for 6 days in separate ex-periments. (**C**,**D**) U937 cells were treated with 5-aza-dC and PJ-34 or combination of these chem-icals for 6 days in separate experiments. The viable cell number was counted. * *p* < 0.05, Mean ± S.E. (**E**,**F**): HL-60 and U937 cells were treated with 5-aza-dC and PJ-34 or a combination of these chemicals and analyzed with flow cytometry by staining of DNA with propidium iodide (PI) at 72 h. SubG1 %, which corresponds to cells with less intensity of PI staining compared to that of G1 phase, is indicated. (**G**) HL-60 cells were treated with 5-aza-dC and PJ-34 or a combination of these chemicals and DNA fragmentation was analyzed at 24 and 72 h.

We used two different blood tumor cell lines which have different oncogenic alterations. HL-60 is a promyelocytic leukemia cell line. PARP inhibitor PJ-34 alone did not show cytotoxicity at 3 µM but showed at 10 µM after 6 days of treatment, while 5-aza-dC exhibited cytotoxicity at 0.5 µM in this cell line. The combinational treatment of 5-aza-dC and PJ-34 exhibited a stronger cytotoxicity compared with their treatment alone. (Figure 1A,B).

For the U937 cells, which are derived from histiocytic lymphoma, treatment with PARP inhibitor alone also showed cytotoxicity at 10 µM after 6 days of treatment. 5-Aza-dC showed cytotoxicity at 0.5 µM in U937. Compared with 5-aza-dC alone, combinational treatment with PJ-34 increased cytotoxicity also in the U937 cells at day 6 (Figure 1C,D).

Flow cytometry analysis of staining DNA with propidium iodide (PI) showed that the subG1 percentage, which corresponds to cells with less intensity of PI staining compared to those of G1 phase, that are induced by DNA degradation during apoptosis, was increased by combinational treatment with PARP inhibitor PJ34 with 5-aza-dC in both HL-60 cells and U937 cells, suggesting apoptosis was increased in these cells (Figure 1E,F). We also observed that increased staining with PI occurred after 5-aza-dC treatment in both HL-60 and U937 cells. Both G1 and G2 peaks were shifted, indicating that PI staining intensity is increased after 5-aza-dC treatment. As 5-aza-dC is a demethylating agent, genomic relaxation by the decreased level of DNA methylation may have caused this increase in the PI staining intensity. Notably, the presence of PARP inhibitor also slightly enhanced the PI staining, suggesting the chromatin state became more relaxed after the combinational treatment. Due to this shifting, it was difficult to analyze the ratio of cell cycle phases.

Analysis of DNA fragmentation by agarose gel electrophoresis (Figure 1G) on day 3 also showed DNA ladder formation after treatment with 5-aza-dC, suggesting typical apoptosis proceeded. On the other hand, after PJ-34 treatment and the combinational treatment, smear larger sized DNA fragments but not nucleosomal DNA ladder appeared, suggesting that the cell death process occurred differently compared with 5-aza-dC treatment after treatment with PJ-34 and the 5-aza-dC/PJ-34 combination. It is considered that apoptosis is mainly responsible for the enhanced cytotoxicity in the 5-aza-dC/PJ-34 combination rather than the growth delay in the HL-60 and U937 cells.

### 3.2. PARP Inhibitor Enhanced Cytotoxicity of 5-aza-dC in Colon Cancer HCT116 and RKO Cell Lines

**Figure 2:**
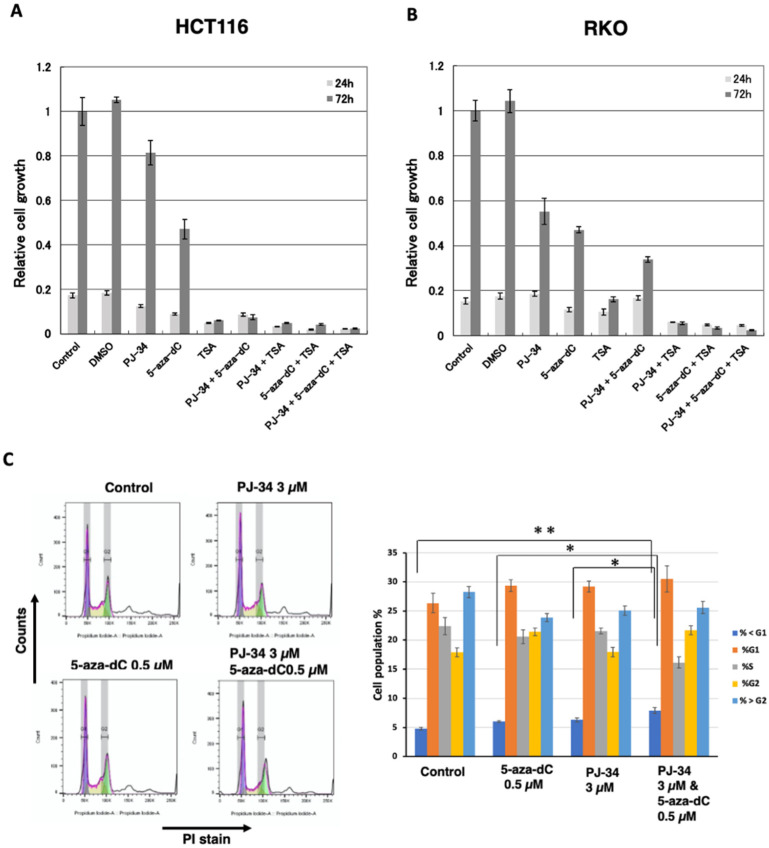
Effects of PJ-34, 5-aza-dC, and TSA in colon cancer HCT116 and RKO cells. (**A**,**B**) Effects of 3 µM PJ-34, 1 µM 5-aza-dC, and 300 nM TSA on the cell growth in colon cancer HCT116 (**A**) and RKO (**B**) for 3 days. *n* = 3. Statistical differences are shown in Table S1. (**C**) HCT116 cells were treated with 5-aza-dC, PARP inhibitors PJ-34 for 2 days and analyzed with flow cytometry analysis. The cells were stained with PI. The left panel shows the representative cell cycle distribution. The right panel shows the cell population % of subG1, G1, S, G2, and superG2 fractions. *N* = 3. Mean ± S.E. Statis-tical differences in SubG1 % is indicated. * *p* < 0.05, ** *p* < 0.01.

To examine whether the combination of PARP inhibitor PJ34 with 5-aza-dC enhances cytotoxicity in solid cancer cells, the effect of PJ-34 and 5-aza-dC was analyzed in two different colon cancer cells HCT116 and RKO for 3 days. We also examined the combination of HDAC inhibitor TSA, which induces histone acetylation and shows cytotoxicity to cancer cells. The combination of 5-aza-dC and HDAC inhibitor was reported to induce the expression of silenced cancer suppressor genes in a coordinated manner [6,7]. As shown in Figure 2A and Table S1, PJ-34 alone did not show cytotoxicity, in contrast, it enhanced cytotoxicity of 5-aza-dC in HCT116 cells (*p* < 0.001). TSA at 300 nM alone showed a high cytotoxicity but combinational treatment with PJ-34, 5-aza-dC or both did not enhance cytotoxicity. In RKO cells, PJ-34 alone did show increased cytotoxicity compared with the control (*p* < 0.001). On the other hand, the enhanced cytotoxicity of 5-aza-dC by the combination (*p* < 0.01) was observed as shown in Figure 2B and Table S1. Also in RKO, TSA at 300 nM alone showed a high cytotoxicity and the combinational treatment with PJ-34 (*p* < 0.05), 5-aza-dC (*p* < 0.01), or both (*p* < 0.001) enhanced the cytotoxicity.

We further analyzed the effect on the cell cycle phase and subG1 apoptotic fractions by flow cytometry after combinational treatment with PJ-34, 5-aza-dC, and single treatments 48 hrs after drug addition. As shown in Figure 2C, the combinational treatment with 5-aza-dC/PJ-34 only slightly increased the subG1 fraction compared to the control or single treatment of 5-aza-dC or PJ-34 without clearly affecting the cell cycle phase ratio in HCT116 cells (Figure 2C). This suggests cell growth delay seemed to contribute to the enhanced cytotoxicity of 5-aza-dC/PJ-34 combination.

We also compared PJ-34 and two clinically used PARP inhibitors, olaparib and talazoparib, for the combination effect on 5-aza-dC in HCT116 cells (Table 1). Olaparib did not show cytotoxicity as in the case with PJ-34, whereas talazoparib showed cytotoxicity in a single treatment. Olaparib and talazoparib also enhanced cytotoxicity of 5-aza-dC in HCT116 cells as observed with PJ-34 as listed in Table 1. As the reported action mechanism of talazoparib was different from other PARP inhibitors [33], in this study we focused on the combination effect of PJ-34 and 5-aza-dC.

### 3.3. Treatment with 5-aza-dC but Not PJ-34 Caused SSBs and Alkali-Labile DNA Damage and Apoptotic DNA Ladder Formation in HCT116 Cells

**Figure 3:**
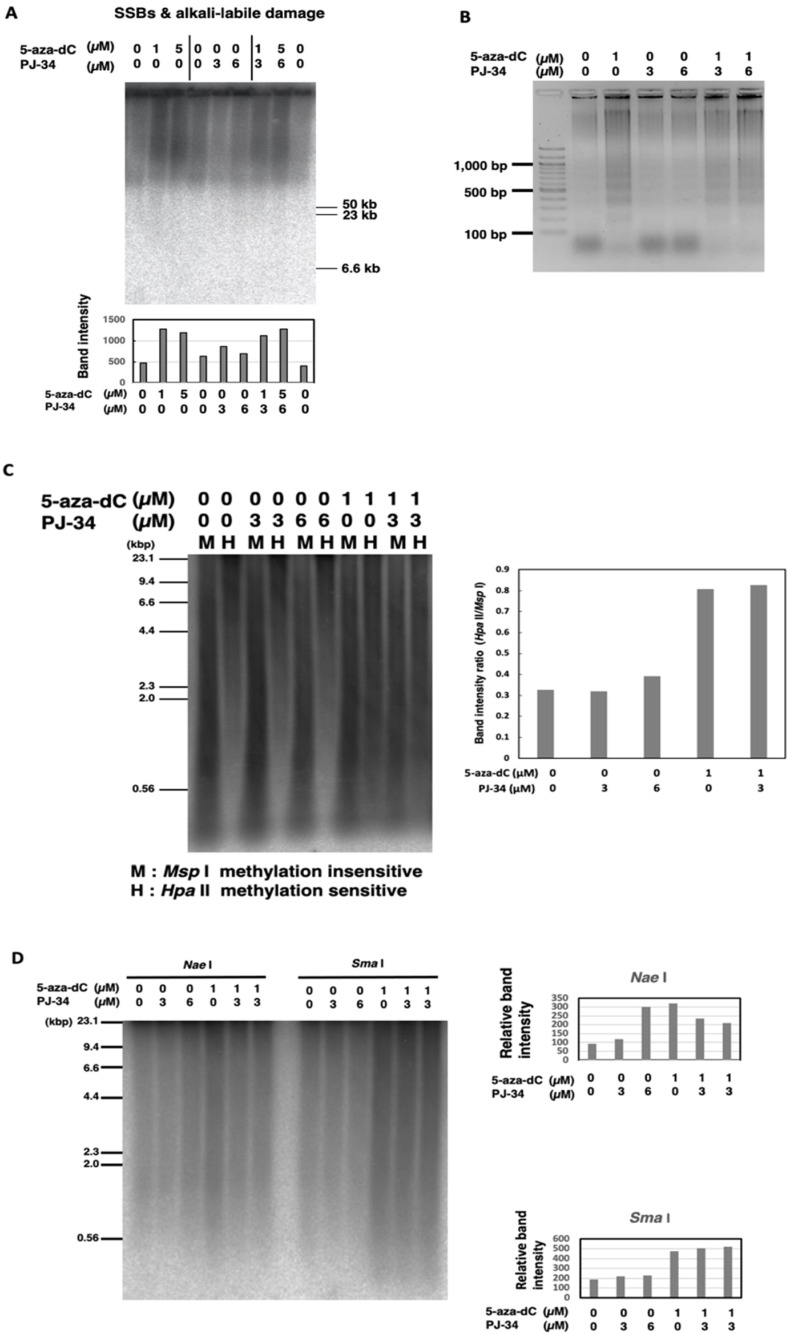
Effect of PJ-34 and 5-aza-dC on SSBs and alkali-labile DNA damage and apoptotic DNA frag-mentation and DNA methylation in colon cancer HCT116 cells. (**A**) The cells were treated with 5-aza-dC, PARP inhibitors PJ-34 for 3 days and genome DNA was analyzed by pulse-field gel electrophoresis in alkaline treatment to observe SSBs and alkali-labile DNA damage and detected with total genome probe by Southern blot analysis. The band intensity was quantified and plotted in the graph shown below. (**B**) Induction of apoptotic DNA ladder formation on day 6 with 5-aza-dC treatment but not with PARP inhibitor. No enhancement of DNA ladder formation was observed with the presence of 5-aza-dC and PARP inhibitor. (**C**,**D**): HCT116 cells were treated with 5-aza-dC, PJ-34, and a combination of 5-aza-dC, PJ-34 for 3 days. Analysis of the DNA methylation level by methylation-insensitive *Msp* I and methylation-sensitive *Hpa* II restriction enzyme digestion (**C**) or methylation sensitive *Nae* I and *Sma* I (**D**). The DNA methylation level was detected with total genome probe by Southern blot analysis. The band intensity was quantified and plotted in the graphs that are shown, respectively.

As 5-aza-dC was reported to cause SSBs [34], we measured SSBs and alkali-labile DNA damage to investigate the mechanism for the enhancement of cytotoxicity by 5-aza-dC/PJ-34 combination in HCT116 cells. SSBs and alkali-labile DNA damage were analyzed by pulse-field gel electrophoresis (PFGE) of genome DNA in alkaline conditions and after being transferred to a membrane, Southern blot hybridization with genomic DNA was carried out [31,32]. SSBs and alkali-labile DNA damage at the range of several ten kb or larger sizes were detected 3 days after treatment with 5-aza-dC at 1 µM as shown in Figure 3A. SSBs and alkali-labile DNA damage were not induced after PJ-34 treatment. The combination treatment of PJ-34 and 5-aza-dC did not increase the level of SSBs and alkali-labile DNA damage compared with 5-aza-dC alone. Apoptotic DNA ladder formation at the range of 200 bp–20 kbp was detected later, 6 days after treatment, with 5-aza-dC at 1 µM but not with PJ-34 at 3 or 6 µM and the combinational treatment did not enhance apoptotic DNA ladder formation (Figure 3B). As shown in Figure 2C, we have analyzed the effect on the cell cycle and detected only a slight increase in the apoptosis fraction on day 2. Therefore, it is suggested that a growth delay rather than apoptosis mainly contributed to the enhanced cytotoxicity under the PJ-34 and 5-aza-dC combination treatment in HCT116 cells.

### 3.4. Global Genome DNA Demethylation was Induced by 5-aza-dC but Not Affected by PJ-34 in HCT116

As 5-aza-dC is a DNMT inhibitor, we analyzed the effect of combinational treatment for 3 days on the genome DNA methylation level by methylation-sensitive restriction enzyme digestions. As shown in Figure 3C, analysis by methylation-insensitive *Msp* I and methylation-sensitive *Hpa* II restriction enzyme digestion showed that 5-aza-dC at 1 µM induced DNA demethylation, while PJ-34 at 3 or 6 µM did not. The combination did not affect DNA demethylation that was induced by 5-aza-dC. We also observed that the treatment with 5-aza-dC at 1 µM or with PJ-34 at 6 µM induced DNA demethylation at *Nae* I site (Figure 3D), whereas DNA demethylation was slightly reduced by their combinational treatment. On the other hand, with *Sma* I digestion, we observed that DNA demethylation was induced by 5-aza-dC but PJ-34 treatment did not affect DNA demethylation (Figure 3D). The results might suggest the possibility that PJ-34 also affects particular DNA methylation states in a DNA sequence context manner. To analyze the effect of PJ-34 from the aspects of transcriptional regulation, we further investigated the transcriptome after treatment with these agents in HCT116 and RKO cells.

### 3.5. Induction of Distinct Changes in Transcriptional Profiles by 5-aza-dC, PARP Inhibitor, and Their Combination in HCT116 and RKO Cells

**Figure 4:**
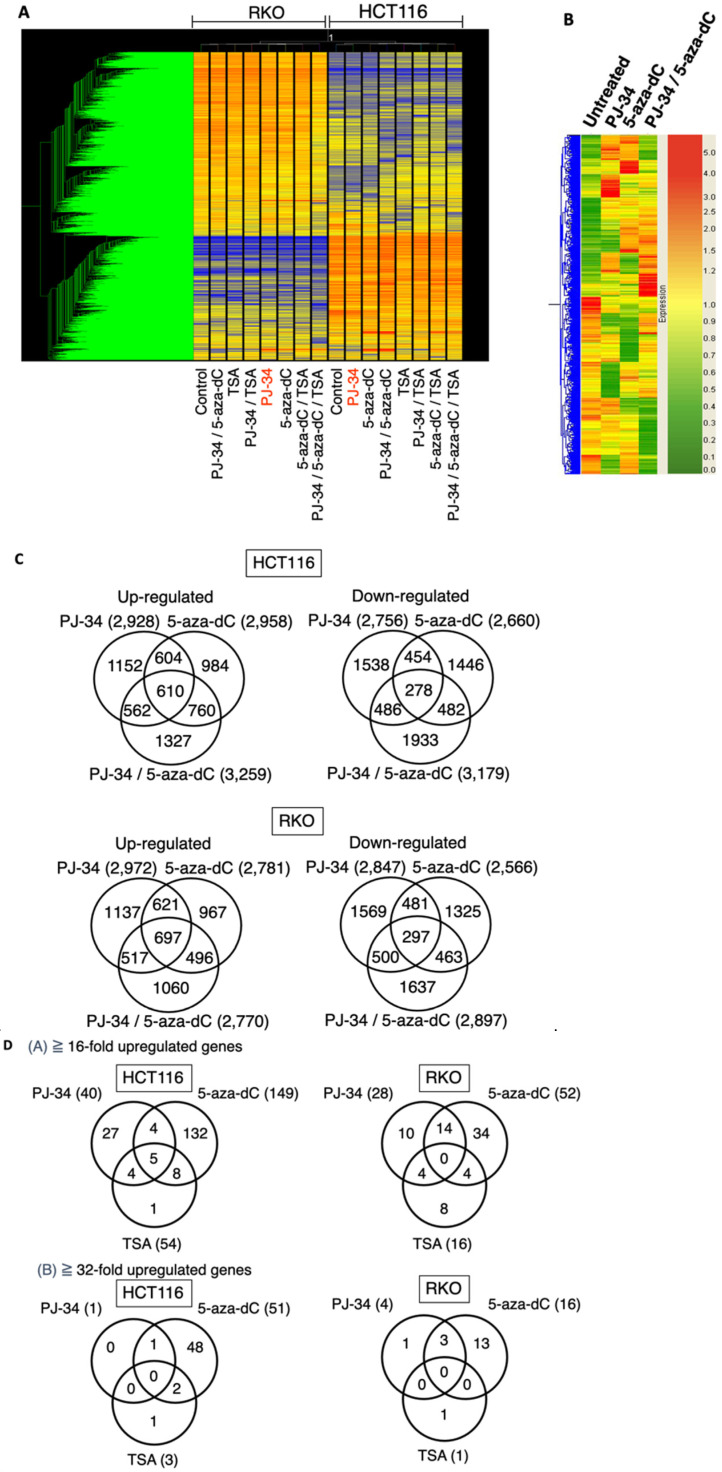

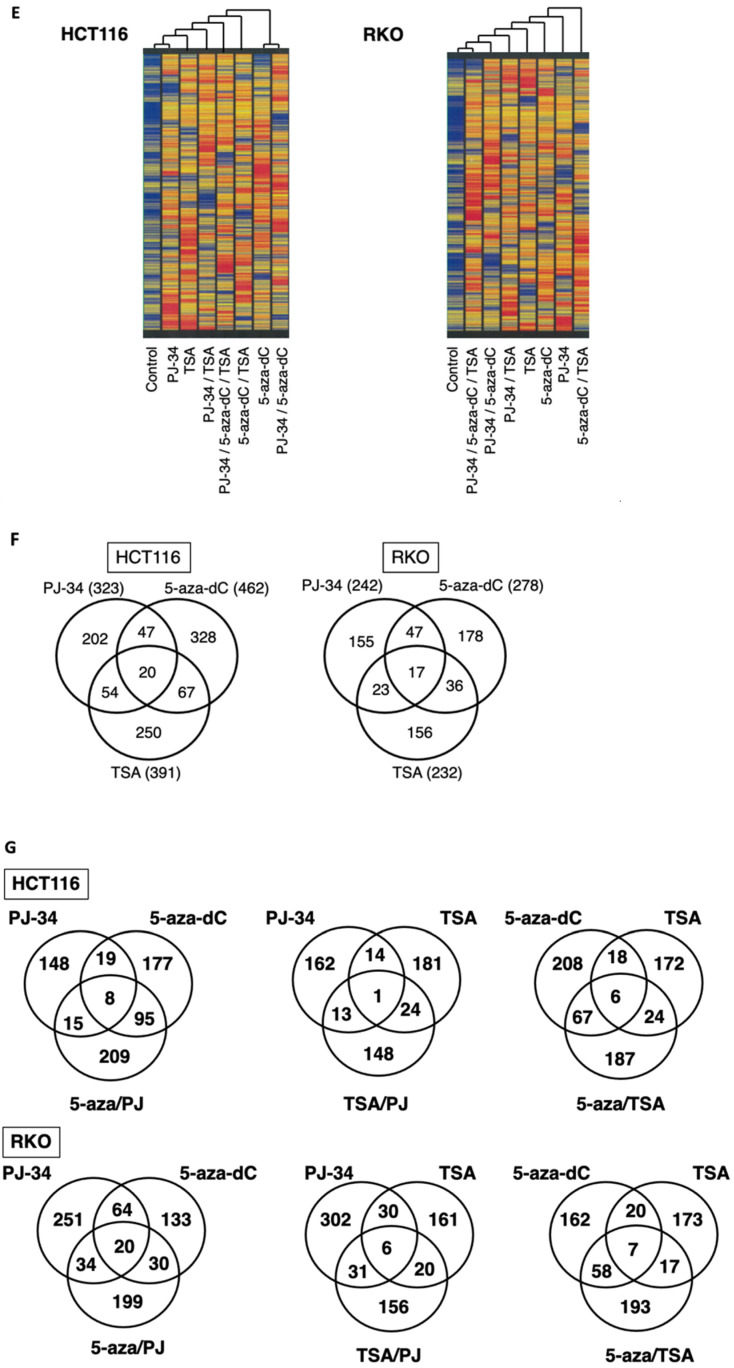
Effect of PJ-34 and 5-aza-dC on the gene expression profiles in HCT116 and RKO cells. HCT116 and RKO cells were treated with 5-aza-dC (5-aza) at 0.5 µM, PJ-34 (PJ) at 3 µM, TSA at 150 nM as a single treatment or in combinations for 3 days and the gene expression profiles were analyzed by microarray analysis. (**A**) Cluster analysis of gene expression profiles in HCT116 and RKO cells. (**B**) Cluster analysis of the gene expression profiles in HCT116 using 12,765 genes chosen. (**C**) Venn diagrams for genes showing 5-fold changes or more compared with the untreated condition in HCT116 and RKO cells. (**D**) Number of genes showing up-regulation by the single treatments with PJ-34, 5-aza-dC, and TSA in HCT116 and RKO cells (**A**) 16-fold or more; (**B**) 32-fold or more). (**E**) Left panel: Cluster analysis of reactivated genes that were induced by PJ3-4, 5-aza-dC, TSA, and their combinations in HCT116 cells (7742 genes chosen). Right panel: Cluster analysis of reactivated genes by PJ-34, 5-aza-dC, TSA, and their combinations in RKO cells (7720 genes chosen). Genes which showed a control level of raw data lower than 50 and showing reactivation that was 5-fold or more to reach at least to raw data of 50 in at least one single treatment condition were used. (**F**) Venn diagrams for the comparison of reactivated genes in HCT116 and RKO cells that were in-duced by the single treatments. (**G**) Venn diagrams for comparison of the reactivated genes in HCT116 and RKO cells that were induced by the single and combinational treatments. Genes of raw data for control less than value 50, and the induction of 5-fold or more to the value of 50 or more, where the flag values of present or marginal are shown. The flag values indicate the quality of signals; present means good, marginal means intermediate, while absent means bad.

The HCT116 and RKO cells were treated with 5-aza-dC at 0.5 µM, PJ-34 at 3 µM, TSA at 150 nM as a single treatment or in combination for 3 days, and the gene expression profiles were analyzed by microarray analysis. In this analysis, we additionally analyzed TSA treatment, because HDAC inhibitor actions in transcriptional profiles are expected to be different from that of 5-aza-dC and their combinational treatments may provide information on action mechanism of PJ-34. As shown in Figure 4A, HCT-116 and RKO exhibited distinct transcriptional profiles in non-treated conditions as well as after drug treatments, probably due to background genetic and epigenomic differences. Therefore, we analyzed these cells separately in the analysis.

In the HCT116 cells, cluster analysis showed that PJ-34, 5-aza-dC, and their combinational treatment showed a distinct pattern of up- and down-regulated gene profiles as shown in Figure 4B. Venn diagrams for the numbers of differently up- and down-regulated genes after treatment with PJ-34, 5-aza-dC, and their combination showed that combinational treatment showed more up- and down-regulated genes than single treatments in HCT116 cells (Figure 4C, upper panels). In the RKO cells, the numbers of differently up- and down-regulated genes after treatment with PJ-34, 5-aza-dC, and their combination were similar (Figure 4C, lower panels).

When we narrowed down to 16-fold and 32-fold or more up-regulated genes in HCT116 and RKO cells after single treatments of PJ-34, 5-aza-dC and TSA as shown in Figure 4D, we found only one and four genes remained as up-regulated genes after PJ-34 treatment in HCT116 and RKO cells, respectively. In the case of TSA, only three and one genes remained as up-regulated genes in HCT116 and RKO cells, respectively. On the other hand, 51 and 16 genes still remained as up-regulated genes after 5-aza-dC treatment in HCT116 and RKO cells, respectively. It may suggest that the potential of gene up-regulation is higher with 5-aza-dC compared with PJ-34 and TSA. In these genes, *PDGFD* [35] was commonly up-regulated by PJ-34 and 5-aza-dC in HCT116 cells. *RANBP9* [36] was commonly up-regulated by PJ-34. *GAGE5* [37] was up-regulated commonly by PJ-34 and 5-aza-dC in RKO cells. These three genes are known to be overexpressed in cancers, suggesting that the up-regulation of these genes may decrease anti-tumor effects.

### 3.6. Distinct Profiles of Reactivation of Silenced Genes by 5-aza-dC, PARP Inhibitor, and Their Combination in HCT116 and RKO Cells

Among the transcriptional profiles, we focused on silenced genes and their reactivation after PJ-34, 5-aza-dC, and the combinational treatment of both drugs in comparison with TSA treatment alone or in combination. In Figure 4E, genes which showed the control level of raw data lower than 50 and showing reactivation to five-fold or more to reach at least to raw data of 50 in at least one single treatment condition were chosen and cluster analysis was carried out in HCT116 and RKO cells. In each cell line, single treatments show different gene reactivation patterns and their combinations also caused distinct reactivation profiles. When we compared the reactivated genes in single treatment conditions, the numbers of commonly reactivated genes were less than differently reactivated genes in HCT-116 and RKO cells (Figure 4F). In differently reactivated genes, *SOCS3* [38] and *SFRP4* [39] as potential tumor suppressor genes, were noted in PJ-34 and 5-aza-dC treatments, respectively. Similarly, *CDX2* and *LOX*, *UCHL1*, and *SHRP1* were found in the reactivated genes of PJ-34 and 5-aza-dC treatments, respectively, in RKO cells. *CDH*1, a tumor suppresor gene which encodes E-cadherin [40], was reactivated commonly in 5-aza-dC and TSA treatment in RKO cells. While 5-aza-dC is reported to reactivate a tumor suppressor *p16INK4A* gene [41], no tumor suppressor candidate gene was found among the commonly reactivated genes in PJ-34, 5-aza-dC, and TSA treatments in the either the HCT116 or RKO cell lines.

Next, we analyzed the differences and commonness in the reactivated genes in the either two drug combinations and their single treatments. As shown in Figure 4G, in both HCT116 and RKO cells, the numbers of reactivated genes were higher in 5-aza-dC/PJ-34 and 5-aza-dC/TSA combinations than TSA/PJ-34 combinations.

From the therapeutic viewpoint, if combinational treatment can reactivate genes at higher levels than in single treatments, the potential therapeutic benefits can be expected. Therefore, we further classified the reactivated genes in HCT116 and RKO cells by the reactivation levels in single or two drug combinations as shown in Table 2. Notably, the combination of either 5-aza-dC/PJ-34, 5-aza-dC/TSA, and TSA/PJ-34 showed numbers of reactivated genes at higher levels than in each single treatment condition (Table 2(i,ii)). Among these two drug combinations, 5-aza-dC/PJ-34 combination showed the highest numbers of reactivated genes that showed reactivation only in the combinational treatment in both HCT116 and RKO cells (Table 2(iii)).

## 4. Discussion

In this study, we investigated the effect of combinational treatment of DNMT inhibitor and PARP inhibitor on the cytotoxicity of cancer cells and found that their combination enhances cytotoxicity in both blood cancer and colon cancer cells. The cytotoxicity of PJ-34 to HL60 and Jarkat cells was previously shown [42], whereas the enhancement of cytotoxicity by combinational treatments of DNA damaging agents, such as doxorubicin, etoposide, cytrabine, and chrolambucil with PJ-34 was not observed [42]. Here in this study, we used 5-aza-dC as DNMT inhibitor and PJ-34 mainly as a PARP inhibitor and demonstrated that their combinational treatment induces distinct changes in the transcriptional profiles and reactivation of different types of silenced genes in single and combinational treatments (Figure 4 and Table 2). The altered gene expression profiles were also different from those that were induced by the HDAC inhibitor TSA or its combination with 5-aza-dC. These results suggest that the combination of PARP inhibitor with 5-aza-dC may be useful as a treatment of cancers.

We observed transcription levels of more than hundreds of different genes were up-regulated and down-regulated in 5-aza-dC- and PJ-34-treated cells and, in their combination, suggesting that broad transcriptional alterations occurred. In the case of 5-aza-dC, because we found SSBs/alkali-labile damage is extensively generated (Figure 3A) as previously reported [34], we speculate that genome-wide demethylation as well as SSBs that are induced by DNA replication blockade may contribute to chromatin relaxation and triggers genome-wide changes, not only the up-regulation but also the down-regulation of gene expression. On the other hand, although the PARP inhibitor is reported to block SSB repair and alter chromatin regulations, we could not detect an increase in SSBs/alkali-labile DNA damage after PJ-34 treatment at 3 or 6 µM in the HCT116 cells (Figure 3A); meanwhile, we observed genome-wide transcriptional changes (Figure 4). PARPs consist of 17 family member proteins and it is a question that the inhibition of which PARP molecule is responsible for these transcriptional changes. When *Parp-1* knockout mice were subjected to transcriptional analysis, global transcriptional changes were reported [43]. PARP-1 and PARP-7 are also shown to be involved in pluripotency control of stem cells from chromatin regulation [19]. PARP-2 [44] and tankyrase [45,46] are reported to be involved in transcriptional and chromatin regulation. The clarification of major target PARP(s) that are responsible for transcriptional regulations may be useful for further translational research of PARP inhibitors to clinical applications.

As more extensive genome-wide changes occurred after combinational treatment with 5-aza-dC and PJ-34 compared with the single treatments (Figure 4), the up-regulation of either tumor suppressor genes or the down-regulation as well as expression changes of oncogenes could possibly affect the enhanced cytotoxicity. As shown, among commonly up-regulated genes of 32-fold or more by PJ-34 and 5-aza-dC treatment, as candidate oncogenic genes, *PDGFD* and *GAGE5* were found, respectively, for HCT116 and RKO cells. Therefore, it was considered difficult to identify the major transcriptional alterations that are responsible for the enhanced cytotoxic actions.

In the case with blood cancer cell lines, we observed that apoptosis was enhanced by the combinational treatment of 5-aza-dC and PJ-34. However, with HCT-116, growth delay seemed to be the main action and apoptosis was only slightly increased at least on days 2–3. Analyzing the time course with gene expression changes will be useful for further elucidation of enhanced cytotoxicity.

In vivo xenograft models of combinational treatment of 5-aza-dC and PJ-34 or with other inhibitors of DNMT and PARP will be also necessary for the further optimization of the treatment conditions. If transcriptional changes and DNA replication block both affect the cytotoxicity that is induced by the combination treatment, it may be also worth finding the optimum treatment order and timing of each drug. We also observed treatment with PJ-34 at 6 µM induced DNA demethylation at *Nae* I site as in the case of 5-aza-dC, although the effect of PJ-34 on global DNA methylation was not detected with digestion at *Hpa* II or *Sma* I. A possible speculation could be that PJ-34 may have affected the DNA methylation level of particular sequences in the genome.

It was reported that base excision repair (BER) factors recognize 5-aza-dC-induced lesions in DNA and the combination of 5-aza-dC and PARP inhibitor may cause a synergistic inhibition of DNA repair to improve treatment [34]. However, in colon cancer cells, we did not observe enhanced DNA damage in the combinational treatment with 5-aza-dC and PARP inhibitor. Therefore, it is suggested that the combination of 5-aza-dC and PARP inhibitor can sensitize at least particular cancer cells by inducing broad and distinct transcriptional changes.

5-Aza-dC has been clinically used under the name decitabine. The efficacy of DNA methylation inhibitor decitabine plus the cytidine deaminase inhibitor cedazuridine in the treatment of MDS and chronic myelomonocytic leukemia was demonstrated in the recent clinical studies of ASTX727-01-B and ASTX727-02 and they were approved by the United States Food and Drug Administration [3,4,5]. The ASTX727-01-B study showed that 18% that were in complete remission and 49% of patients were free from transfusion dependence, and the ASTX727-02 study showed that 21%that were in complete remission and 53% of patients were free from transfusion dependence. The ASTX727-02 trial showed that 21% that were in complete remission and 53% of patients were free from transfusion dependency [3,4,5]. These data suggest that the improvement of therapeutic strategy is still necessary.

A Phase I clinical trial reported some efficacy of decitabine and PARP inhibitor talazoparib combination therapy in relapsed/refractory AML and further studies are warranted [47]. Azacytidine is also an inhibitor of DNMT and is similarly incorporated into DNA as 5-aza-dC. Azacytidine has been tested as a single agent as well in the treatment of high-risk MDS. The CALGB9221 trial showed prolonged time to leukemic transformation, improved overall survival, and quality of life [48]. The AZA001 trial showed that the time to leukemic transformation was prolonged and the overall survival was improved [49]. It will be also worth studying whether the combinational treatment of azacytidine and PARP inhibitors sensitize clinical cancers.

## 5. Conclusions

Taken together, these results suggest that combinational treatment with 5-aza-dC and PARP inhibitor could be useful by changing global transcriptional profiles from the profiles of single treatments. These studies will help us understand the involvement of poly(ADP-ribosylation) in the regulation of cancer cell proliferation, epigenome regulation, and cell death, and will enable new applications of poly(ADP-ribosylation)-targeted drugs in cancer therapy.

## Figures and Tables

**Table 1:** The IC50 and IC30 values of 5-aza-dC, PARP inhibitors, and their combinations for HCT116 cells.

Drug	IC50 *	IC30 *
5-aza-dC	>0.7 μM	0.47 μM
PJ-34	>3.4 μM	>3.4 μM
5-aza-dC/PJ-34 (7:34) ^#^	5-aza-dC 0.61 μM	5-aza-dC 0.44 μM
	PJ-34 3.0 μM	PJ-34 2.1 μM
5-aza-dC	>0.7 μM	0.57 μM
Olaparib	>3.8 μM	3.3 μM
5-aza-dC/Olaparib (7:38) ^#^	5-aza-dC 0.28 μM	5-aza-dC 0.13 μM
	Olaparib 1.5 μM	Olaparib 0.74 μM
5-aza-dC	>0.7	0.61
Talazoparib	0.095	0.032
5-aza-dC/Talazoparib (7:1) ^#^	5-aza-dC 0.14 μM	5-aza-dC 0.64 μM
	Talazoparib 0.02 μM	Talazoparib 0.0092 μM

* CCK assay was carried out 4 days after treatment. ^#^ Molar ratio. For this assay, the ratios of the drugs in the combination treatment were fixed, respectively. IC50-IC30 values for single treatments were chosen for combinational treatment where possible.

**Table 2:** Number of genes that were reactivated by the combinational treatment of chemicals, where flag shows present or marginal.

(i) Both single and combinational treatments show reactivation, and the combinational treatment shows higher levels of reactivation than the reactivation by the single treatment.		
Combination	HCT116	RKO
PJ-34/5-aza-dC	48	51
PJ-34/TSA	29	31
5-aza-dC/TSA	51	45
(ii) Either of single treatments shows reactivation, and the combinational treatment shows higher levels of reactivation than the reactivation by the single treatment		
Combination (classified in groups with the level of reactivation in single treatment)	HCT116	RKO
PJ-34 > 5-aza-dC	35	29
5-aza-dC > PJ-34	31	89
PJ-34 > TSA	43	23
TSA > PJ-34	27	33
5-aza-dC > TSA	56	26
TSA > 5-aza-dC	33	33
(iii) The combinational treatment only shows reactivation		
Combination	HCT116	RKO
PJ-34/5-aza-dC	94	92
PJ-34/TSA	59	47
5-aza-dC/TSA	77	79

## Data Availability

The microarray data is reposited in the GEO database (GSE211027) and the excel file is attached as a Supplementary Table (Table S2).

## Supplementary Materials

The following supporting information can be downloaded at: https://www.mdpi.com/2072-6694/14/17/4171/s1. Table S1: *p*-values calculated by statistical analysis with Tukey’s test for Figure 2A,B. Table S2: The Excel file of microarray data.

## References

[1] Jaenisch R., Bird A. (2003). Epigenetic regulation of gene expression: How the genome integrates intrinsic and environmental signals. Nat. Genet..

[2] Ushijima T. (2007). Epigenetic field for cancerization. J. Biochem. Mol. Biol..

[3] Garcia-Manero G., Griffiths E.A., Steensma D.P., Roboz G.J., Wells R., McCloskey J., Odenike O., DeZern A.E., Yee K., Busque L. (2020). Oral cedazuridine/decitabine for mds and cmml: A phase 2 pharmacokinetic/pharmacodynamic randomized crossover study. Blood.

[4] Kim N., Norsworthy K.J., Subramaniam S., Chen H., Manning M.L., Kitabi E., Earp J., Ehrlich L.A., Okusanya O.O., Vallejo J. (2022). Fda approval summary: Decitabine and cedazuridine tablets for myelodysplastic syndromes. Clin. Cancer Res..

[5] Xu K., Hansen E. (2021). Novel agents for myelodysplastic syndromes. J. Oncol. Pharm. Pract..

[6] Shi H., Wei S.H., Leu Y.W., Rahmatpanah F., Liu J.C., Yan P.S., Nephew K.P., Huang T.H. (2003). Triple analysis of the cancer epigenome: An integrated microarray system for assessing gene expression, DNA methylation, and histone acetylation. Cancer Res..

[7] Yamashita K., Upadhyay S., Osada M., Hoque M.O., Xiao Y., Mori M., Sato F., Meltzer S.J., Sidransky D. (2002). Pharmacologic unmasking of epigenetically silenced tumor suppressor genes in esophageal squamous cell carcinoma. Cancer Cell.

[8] Luszczek W., Cheriyath V., Mekhail T.M., Borden E.C. (2010). Combinations of DNA methyltransferase and histone deacetylase inhibitors induce DNA damage in small cell lung cancer cells: Correlation of resistance with ifn-stimulated gene expression. Mol. Cancer Ther..

[9] Ahrens T.D., Timme S., Hoeppner J., Ostendorp J., Hembach S., Follo M., Hopt U.T., Werner M., Busch H., Boerries M. (2015). Selective inhibition of esophageal cancer cells by combination of hdac inhibitors and azacytidine. Epigenetics.

[10] Schreiber V., Dantzer F., Ame J.C., de Murcia G. (2006). Poly(adp-ribose): Novel functions for an old molecule. Nat. Rev. Mol. Cell Biol..

[11] Kanai M., Tong W.M., Sugihara E., Wang Z.Q., Fukasawa K., Miwa M. (2003). Involvement of poly(adp-ribose) polymerase 1 and poly(adp-ribosyl)ation in regulation of centrosome function. Mol. Cell. Biol..

[12] Fong P.C., Boss D.S., Yap T.A., Tutt A., Wu P., Mergui-Roelvink M., Mortimer P., Swaisland H., Lau A., O’Connor M.J. (2009). Inhibition of poly(adp-ribose) polymerase in tumors from brca mutation carriers. N. Engl. J. Med..

[13] Yasukawa M., Fujihara H., Fujimori H., Kawaguchi K., Yamada H., Nakayama R., Yamamoto N., Kishi Y., Hamada Y., Masutani M. (2016). Synergetic effects of parp inhibitor azd2281 and cisplatin in oral squamous cell carcinoma in vitro and in vivo. Int. J. Mol. Sci..

[14] Ikejima M., Noguchi S., Yamashita R., Ogura T., Sugimura T., Gill D.M., Miwa M. (1990). The zinc fingers of human poly(adp-ribose) polymerase are differentially required for the recognition of DNA breaks and nicks and the consequent enzyme activation. Other structures recognize intact DNA. J. Biol. Chem..

[15] Thomas C., Ji Y., Wu C., Datz H., Boyle C., MacLeod B., Patel S., Ampofo M., Currie M., Harbin J. (2019). Hit and run versus long-term activation of parp-1 by its different domains fine-tunes nuclear processes. Proc. Natl. Acad. Sci. USA.

[16] Cohen-Armon M., Visochek L., Rozensal D., Kalal A., Geistrikh I., Klein R., Bendetz-Nezer S., Yao Z., Seger R. (2007). DNA-independent parp-1 activation by phosphorylated erk2 increases elk1 activity: A link to histone acetylation. Mol. Cell.

[17] Idogawa M., Yamada T., Honda K., Sato S., Imai K., Hirohashi S. (2005). Poly(adp-ribose) polymerase-1 is a component of the oncogenic t-cell factor-4/beta-catenin complex. Gastroenterology.

[18] Gadad S.S., Camacho C.V., Malladi V., Hutti C.R., Nagari A., Kraus W.L. (2021). Parp-1 regulates estrogen-dependent gene expression in estrogen receptor alpha-positive breast cancer cells. Mol. Cancer Res..

[19] Roper S.J., Chrysanthou S., Senner C.E., Sienerth A., Gnan S., Murray A., Masutani M., Latos P., Hemberger M. (2014). Adp-ribosyltransferases parp1 and parp7 safeguard pluripotency of es cells. Nucleic Acids Res..

[20] Yu W., Ginjala V., Pant V., Chernukhin I., Whitehead J., Docquier F., Farrar D., Tavoosidana G., Mukhopadhyay R., Kanduri C. (2004). Poly(adp-ribosyl)ation regulates ctcf-dependent chromatin insulation. Nat. Genet..

[21] Pavri R., Lewis B., Kim T.K., Dilworth F.J., Erdjument-Bromage H., Tempst P., de Murcia G., Evans R., Chambon P., Reinberg D. (2005). Parp-1 determines specificity in a retinoid signaling pathway via direct modulation of mediator. Mol. Cell.

[22] Hassa P.O., Haenni S.S., Buerki C., Meier N.I., Lane W.S., Owen H., Gersbach M., Imhof R., Hottiger M.O. (2005). Acetylation of poly(adp-ribose) polymerase-1 by p300/creb-binding protein regulates coactivation of nf-kappab-dependent transcription. J. Biol. Chem..

[23] Tulin A., Spradling A. (2003). Chromatin loosening by poly(adp)-ribose polymerase (parp) at drosophila puff loci. Science.

[24] Osada T., Ryden A.M., Masutani M. (2013). Poly(adp-ribosylation) regulates chromatin organization through histone h3 modification and DNA methylation of the first cell cycle of mouse embryos. Biochem. Biophys. Res. Commun..

[25] Osada T., Nozaki T., Masutani M. (2016). Parp1 deficiency confers defects in chromatin surveillance and remodeling during reprogramming by nuclear transfer. Curr. Protein Pept. Sci..

[26] Sun C., Fang Y., Yin J., Chen J., Ju Z., Zhang D., Chen X., Vellano C.P., Jeong K.J., Ng P.K. (2017). Rational combination therapy with parp and mek inhibitors capitalizes on therapeutic liabilities in ras mutant cancers. Sci. Transl. Med..

[27] Cameron E.E., Bachman K.E., Myöhänen S., Herman J.G., Baylin S.B. (1999). Synergy of demethylation and histone deacetylase inhibition in the re-expression of genes silenced in cancer. Nat. Genet..

[28] Fuks F., Hurd P.J., Wolf D., Nan X., Bird A.P., Kouzarides T. (2003). The methyl-cpg-binding protein mecp2 links DNA methylation to histone methylation. J. Biol. Chem..

[29] Zhang Y., Fatima N., Dufau M.L. (2005). Coordinated changes in DNA methylation and histone modifications regulate silencing/derepression of luteinizing hormone receptor gene transcription. Mol. Cell. Biol..

[30] Zampieri M., Guastafierro T., Calabrese R., Ciccarone F., Bacalini M.G., Reale A., Perilli M., Passananti C., Caiafa P. (2012). Adp-ribose polymers localized on ctcf-parp1-dnmt1 complex prevent methylation of ctcf target sites. Biochem. J..

[31] Sutherland J.C., Monteleone D.C., Mugavero J.H., Trunk J. (1987). Unidirectional pulsed-field electrophoresis of single- and double-stranded DNA in agarose gels: Analytical expressions relating mobility and molecular length and their application in the measurement of strand breaks. Anal. Biochem..

[32] Freeman S.E., Blackett A.D., Monteleone D.C., Setlow R.B., Sutherland B.M., Sutherland J.C. (1986). Quantitation of radiation-, chemical-, or enzyme-induced single strand breaks in nonradioactive DNA by alkaline gel electrophoresis: Application to pyrimidine dimers. Anal. Biochem..

[33] Palve V., Knezevic C.E., Bejan D.S., Luo Y., Li X., Novakova S., Welsh E.A., Fang B., Kinose F., Haura E.B. (2022). The non-canonical target parp16 contributes to polypharmacology of the parp inhibitor talazoparib and its synergy with wee1 inhibitors. Cell Chem. Biol..

[34] Orta M.L., Höglund A., Calderón-Montaño J.M., Domínguez I., Burgos-Morón E., Visnes T., Pastor N., Ström C., López-lázaro M., Helleday T. (2014). The parp inhibitor olaparib disrupts base excision repair of 5-aza-2’-deoxycytidine lesions. Nucleic Acids Res..

[35] Chen J., Yuan W., Wu L., Tang Q., Xia Q., Ji J., Liu Z., Ma Z., Zhou Z., Cheng Y. (2017). Pdgf-d promotes cell growth, aggressiveness, angiogenesis and emt transformation of colorectal cancer by activation of notch1/twist1 pathway. Oncotarget.

[36] Qin C., Zhang Q., Wu G. (2019). Ranbp9 suppresses tumor proliferation in colorectal cancer. Oncol. Lett..

[37] Duan Z., Duan Y., Lamendola D.E., Yusuf R.Z., Naeem R., Penson R.T., Seiden M.V. (2003). Overexpression of mage/gage genes in paclitaxel/doxorubicin-resistant human cancer cell lines. Clin. Cancer Res..

[38] Dong X., Wang J., Tang B., Hao Y.X., Li P.Y., Li S.Y., Yu P.W. (2018). The role and gene expression profile of socs3 in colorectal carcinoma. Oncotarget.

[39] Suzuki H., Watkins D.N., Jair K.W., Schuebel K.E., Markowitz S.D., Chen W.D., Pretlow T.P., Yang B., Akiyama Y., Van Engeland M. (2004). Epigenetic inactivation of sfrp genes allows constitutive wnt signaling in colorectal cancer. Nat. Genet..

[40] Christou N., Perraud A., Blondy S., Jauberteau M.O., Battu S., Mathonnet M. (2017). E-cadherin: A potential biomarker of colorectal cancer prognosis. Oncol. Lett..

[41] Fang J.Y., Yang L., Zhu H.Y., Chen Y.X., Lu J., Lu R., Cheng Z.H., Xiao S.D. (2004). 5-aza-2′-deoxycitydine induces demethylation and up-regulates transcription of p16ink4a gene in human gastric cancer cell lines. Chin. Med. J. (Engl.).

[42] Stepnik M., Spryszynska S., Gorzkiewicz A., Ferlinska M. (2017). Cytotoxicity of anticancer drugs and pj-34 (poly(adp-ribose)polymerase-1 (parp-1) inhibitor) on hl-60 and jurkat cells. Adv. Clin. Exp. Med..

[43] Ogino H., Nozaki T., Gunji A., Maeda M., Suzuki H., Ohta T., Murakami Y., Nakagama H., Sugimura T., Masutani M. (2007). Loss of parp-1 affects gene expression profile in a genome-wide manner in es cells and liver cells. BMC Genomics.

[44] Liang Y.C., Hsu C.Y., Yao Y.L., Yang W.M. (2013). Parp-2 regulates cell cycle-related genes through histone deacetylation and methylation independently of poly(adp-ribosyl)ation. Biochem. Biophys. Res. Commun..

[45] Tang B., Luo H., Xie S., Pan C., Fu J. (2022). Deubiquitination of tnks1 regulates wnt/beta-catenin to affect the expression of usp25 to promote the progression of glioma. Dis. Markers.

[46] Wu Q., Xuan Y.F., Su A.L., Bao X.B., Miao Z.H., Wang Y.Q. (2022). Tnks inhibitors potentiate proliferative inhibition of bet inhibitors via reducing beta-catenin in colorectal cancer cells. Am. J. Cancer Res..

[47] Baer M.R., Kogan A.A., Bentzen S.M., Mi T., Lapidus R.G., Duong V.H., Emadi A., Niyongere S., O’Connell C.L., Youngblood B.A. (2022). Phase i clinical trial of DNA methyltransferase inhibitor decitabine and parp inhibitor talazoparib combination therapy in relapsed/refractory acute myeloid leukemia. Clin. Cancer Res..

[48] Silverman L.R., Demakos E.P., Peterson B.L., Kornblith A.B., Holland J.C., Odchimar-Reissig R., Stone R.M., Nelson D., Powell B.L., DeCastro C.M. (2002). Randomized controlled trial of azacitidine in patients with the myelodysplastic syndrome: A study of the cancer and leukemia group b. J. Clin. Oncol..

[49] Fenaux P., Mufti G.J., Hellstrom-Lindberg E., Santini V., Finelli C., Giagounidis A., Schoch R., Gattermann N., Sanz G., List A. (2009). Efficacy of azacitidine compared with that of conventional care regimens in the treatment of higher-risk myelodysplastic syndromes: A randomised, open-label, phase iii study. Lancet Oncol..

