# Supplementary material for: Enhanced Cytotoxicity on Cancer Cells by Combinational Treatment of PARP Inhibitor and 5-Azadeoxycytidine Accompanying Distinct Transcriptional Profiles
Source: Cancers (Basel). 2022 Aug 28;14(17):4171. doi: 10.3390/cancers14174171 (PMC9454563; doi:10.3390/cancers14174171)
Supplement: Supplementary file 1 [file cancers-14-04171-s001.zip › Table S1 R1.pdf]

Supplemental Table S1. *P* values calculated by statistical analysis with Tukey's test for Figure 2A & B.

HCT116 day 1

	No treatment	PBS	PJ-34	5-aza-dC	TSA	PJ-34 + 5-aza-dC	PJ-34 + TSA	5-aza-dC + TSA	PJ-34 + 5-aza-dC + TSA
No treatment									
PBS	0.4829								
PJ-34	<.0001	<.0001							
5-aza-dC	<.0001	<.0001	<.0001						
TSA	<.0001	<.0001	<.0001	<.0001					
PJ-34 + 5-aza-dC	<.0001	<.0001	<.0001	0.9999	<.0001				
PJ-34 + TSA	<.0001	<.0001	<.0001	<.0001	0.1345	<.0001			
5-aza-dC + TSA	<.0001	<.0001	<.0001	<.0001	0.0004	<.0001	0.1559		
PJ-34 + 5-aza-dC + TSA	<.0001	<.0001	<.0001	<.0001	0.0014	<.0001	0.3996	0.9993	

HCT116 day 3

	No treatment	PBS	PJ-34	5-aza-dC	TSA	PJ-34 + 5-aza-dC	PJ-34 + TSA	5-aza-dC + TSA	PJ-34 + 5-aza-dC + TSA
No treatment									
PBS	0.5528								
PJ-34	<.0001	<.0001							
5-aza-dC	<.0001	<.0001	<.0001						
TSA	<.0001	<.0001	<.0001	<.0001					
PJ-34 + 5-aza-dC	<.0001	<.0001	<.0001	<.0001	0.9997				
PJ-34 + TSA	<.0001	<.0001	<.0001	<.0001	1	0.9395			
5-aza-dC + TSA	<.0001	<.0001	<.0001	<.0001	0.9985	0.9852	1		
PJ-34 + 5-aza-dC + TSA	<.0001	<.0001	<.0001	<.0001	0.899	0.6191	0.9865	0.9985	

RKO day 1

	No treatment	PBS	PJ-34	5-aza-dC	TSA	PJ-34 + 5-aza-dC	PJ-34 + TSA	5-aza-dC + TSA	PJ-34 + 5-aza-dC + TSA
No treatment									
PBS	0.205								
PJ-34	0.0223	0.9511							
5-aza-dC	0.0044	<.0001	<.0001						
TSA	0.0002	<.0001	<.0001	0.8797					
PJ-34 + 5-aza-dC	0.7834	0.965	0.3961	0.0002	<.0001				
PJ-34 + TSA	<.0001	<.0001	<.0001	<.0001	0.0008	<.0001			
5-aza-dC + TSA	<.0001	<.0001	<.0001	<.0001	<.0001	<.0001	0.8328		
PJ-34 + 5-aza-dC + TSA	<.0001	<.0001	<.0001	<.0001	<.0001	<.0001	0.6535	1	

RKO day 3

	No treatment	PBS	PJ-34	5-aza-dC	TSA	PJ-34 + 5-aza-dC	PJ-34 + TSA	5-aza-dC + TSA	PJ-34 + 5-aza-dC + TSA
No treatment									
PBS	0.7347								
PJ-34	<.0001	<.0001							
5-aza-dC	<.0001	<.0001	0.0786						
TSA	<.0001	<.0001	<.0001	<.0001					
PJ-34 + 5-aza-dC	<.0001	<.0001	<.0001	0.0013	<.0001				
PJ-34 + TSA	<.0001	<.0001	<.0001	<.0001	0.0115	<.0001			
5-aza-dC + TSA	<.0001	<.0001	<.0001	<.0001	0.0019	<.0001	0.9925		
PJ-34 + 5-aza-dC + TSA	<.0001	<.0001	<.0001	<.0001	0.0009	<.0001	0.937	1	
